# Antioxidant, Anti-Obesity, and Anti-Aging Activities of *Jeju* Citrus Blended Vinegar

**DOI:** 10.3390/foods10071441

**Published:** 2021-06-22

**Authors:** Ye-Rang Yun, Bo-Yeon Park, Sung-Hyun Kim, Ji-Hye Jung

**Affiliations:** 1 Industrial Technology Research Group, Research and Development Division, World Institute of Kimchi, Nam-Gu, Gwangju 61755, Korea; yunyerang@wikim.re.kr; 2 Hygienic Safety and Analysis Center, Research and Development Division, World Institute of Kimchi, Nam-Gu, Gwangju 61755, Korea; boyeonpark@wikim.re.kr; 3 SME Service Department, Strategy and Planning Division, World Institute of Kimchi, Nam-Gu, Gwangju 61755, Korea; shkim@wikim.re.kr

**Keywords:** citrus blended vinegar, mandarin, premature mandarin, antioxidant, anti-aging, anti-obesity

## Abstract

Various types of vinegars have been developed as interest in their health benefits has increased. In this study, we prepared *Jeju* citrus blended vinegars (CBVs) by mixing premature mandarin vinegar and mandarin vinegar, with mandarin vinegar used as a control. The physicochemical properties of the vinegars, including pH, total acidity, and sugar content was determined. Moreover, antioxidant, anti-obesity, and anti-aging activities of the vinegars were investigated. Physicochemical analysis revealed that the CBVs had a pH similar to that of mandarin vinegar, whereas CBVs with relatively high premature mandarin vinegar content showed higher acidity and lower sugar content (*p* < 0.05). Moreover, the antioxidant activities and phenol contents of CBVs were significantly higher than those of mandarin vinegar (*p* < 0.05). Meanwhile, CBVs showed significantly decreased intracellular triglyceride, lipid accumulation, and anti-obesity related gene levels (*p* < 0.05), thereby highlighting their anti-obesity activity. In addition, CBVs showed anti-aging activity by increasing cell viability and cell lifespan, while decreasing the expression of senescence-related genes under H_2_O_2_-induced oxidative stress. Therefore, CBVs may be useful as a functional food with antioxidant, anti-obesity, and anti-aging effects in various food fields.

## 1. Introduction

Vinegar is a well-known fermented food product that commonly contains 5–8% acetic acid and trace chemicals. Vinegar is typically produced in a two-step fermentation process [1]. Initially, the sugars contained in the source material are converted to alcohol by yeast, which is then exposed to oxygen and fermented with acetic acid bacteria, leading to the generation of vinegar [2]. Recently, blended vinegar has been widely developed and evaluated. Blending is a widespread technique employed for the production of fermented foods, such as wine and vinegar, to improve the sensory properties and functionality of the final product by mixing of different varieties [3,4,5]. For instance, sugarcane blended apple vinegar has been shown to have higher nutritional and antioxidant activities compared with apple vinegar alone [3]. According to Zhang et al., coating ready-to-cook pork chops with chitosan blended bamboo vinegar has antioxidant and antimicrobial effects [4]. Thus, blended vinegar is expected to increase nutritional contents of food and improve their health-associated benefits.

Antioxidant [6], anti-diabetic [7], anti-obesity [8], anti-hypertensive [9], and cholesterol-lowering effects [10] have been reported as beneficial health effects of vinegar. For instance, Budak and Guzel-Seydim reported that the oxygen radical absorbance capacity and Trolox equivalent antioxidant capacity of traditional vinegar were higher than those of commercial vinegar [6]. Moreover, in a study of the anti-obesity effects of vinegar, intake of apple cider vinegar reduced lipid levels in subjects with high-fat diet-induced steatosis [8]. Based on these studies, citrus blended vinegars (CBVs) may have similar health effects.

Citrus fruits, such as mandarin, orange, lime, and lemon, are well known for their various beneficial health properties including antioxidant [11] and anti-obesity effects [12]. In South Korea, mandarin (*Citrus unshiu*) is grown on Jeju Island and is the most widely consumed citrus fruit. Most mandarins are harvested when ripe; however, both the cultivation and consumption of premature fruits have increased in recent years, as premature mandarin contains higher levels of dietary fiber, organic acids, polyphenols, and flavonoids [13]. Indeed, various citrus vinegars have been reported; however, most of these studies have focused on microbial fermentation [14] with only a single study [15] using mandarin as a raw material. Hence, few studies on CBV have been reported.

In this study, we developed CBVs with different blending ratios based on mandarin vinegar (MV). We aimed to investigate whether CBVs exhibit antioxidant, anti-aging, and anti-obesity activities. Initially, we investigated their physicochemical characteristics such as pH, total acidity, and sugar content. Antioxidant activities were also analyzed. Moreover, 3T3-L1 and WI-38 cells were employed to assess the anti-obesity and anti-aging activities, respectively.

## 2. Materials and Methods

### 2.1. Materials

Mandarin, premature mandarin, mandarin concentrates, and premature mandarin concentrates were purchased from local markets (Jeju Island, Korea). Their properties were as follows: mandarin, 12–13° Brix, pH 4.5–4.8; premature mandarin, 7–8° Brix, pH 3.7–3.9; mandarin concentrates, 60° Brix, pH 4.4–4.6; premature mandarin concentrates, 60° Brix, pH 3.4–3.5. The KCCM11304 strain of *Saccharomyces cerevisiae* was obtained from the Korean Culture Center of Microorganisms (Seoul, Korea), and KFCC11858P *Acetobacter pasteurianus* was obtained from the registered strains of the Korean Federation of Culture Collections (Seoul, Korea). Folin–Ciocalteu’s phenol reagent, gallic acid, vitamin C, oil red O (ORO) solution, 3-isobutyl-1-methylxanthine (IBMX), dexamethasone, insulin, chloroform, methanol, ethanol, and formalin were purchased from Sigma Aldrich (St. Louis, MO, USA). Dulbecco’s modified Eagle’s medium (DMEM) and Dulbecco’s phosphate-buffered saline (DPBS) were purchased from Welgen Inc. (Daegu, Korea). Fetal calf serum, fetal bovine serum (FBS), and penicillin/streptomycin were purchased from GIBCO (Invitrogen, Detroit, MI, USA). Na_2_CO_3_ was purchased from Duksan Pure Chemical Co., Ltd. (Ansan, Korea). The cell counting kit-8 (CCK-8) was purchased from Dojindo (Kumamoto, Japan). Finally, the TOPScript^TM^ cDNA synthesis kit and TOPreal^TM^ qPCR 2X PreMIX (SYBR Green with low ROX) were purchased from Enzynomics (Seoul, Korea).

### 2.2. Preparation of CBVs

CBVs were prepared by mixing MV and premature mandarin vinegar (PMV) in proportions. Both MV and PMV were prepared in a two-step fermentation process (alcohol and acetic acid) as follows. Mandarin and premature mandarin were washed with running water and then crushed to extract the juice. In MV, 66.7% juice and 33.3% mandarin concentrate were mixed, whereas PMV was mixed at 55.6% and 44.4%, with a final 24° Brix sugar content. *Saccharomyces cerevisiae* was inoculated at 5% (*v*/*v*), and alcohol fermentation was performed at 25–26 °C for 7 days. After alcohol fermentation, mandarin and premature mandarin wines were filtered (180 mesh), and their alcohol content was diluted to 7% (*v*/*v*). Subsequently, 10% (*v*/*v*) of the *A. pasteurianus* was inoculated in diluted wines, cultured at 120 rpm and 29–30 °C for 15 days, and then filtered (0.22 µm). CBVs were prepared by blending MV and PMV in a ratio of 8:2 (CBV1) or 7:3 (CBV2) and then stored at 4 °C until use.

### 2.3. Analysis of Physiochemical Characteristics of CBVs

The pH of the samples was measured with a pH meter (TitroLine 5000; SI Analytics GmbH, Mainz, Germany); the samples were titrated with 0.1 N NaOH solution until a pH of 8.35 was reached. The titration value (mL) was converted to the acetic acid content (%). The sugar content was measured with a digital sugar meter (SCM-1000, Hm Digital, Seoul, Korea). Alcohol content was calculated by centrifuging (7989× *g*, 15 min, 4 °C) the wine and vinegar samples, distilling 100 mL of the supernatant, and measuring with a hydrometer (211-DK-12, Daekwang, Seoul, Korea) at 15 °C; the value was converted to the alcohol content by using the Gay Lussac Table.

### 2.4. Analysis of Anti-Oxidant Activities of CBVs

#### 2.4.1. Total Phenol Content Analysis

The total phenol content (TPC) was measured using the Folin–Denis method [16]. Samples were initially mixed with Folin–Ciocalteu’s phenol reagent for 1 min, after which 5% Na_2_CO_3_ was added. The samples were reacted in the dark for 1 h and absorbance was measured at 725 nm (SPECTROstar Nano, BMG Labtech, Ortenberg, Germany). The TPC value was expressed as 1 μg of gallic acid equivalent per mL of sample (μg GAE/mL) based on a calibration curve prepared using gallic acid as a standard.

#### 2.4.2. Analysis of 2,2-Diphenyl-1-Picrylhydrazyl (DPPH) Radical Scavenging Activity

The DPPH radical scavenging activity was measured as described previously [17]. Samples were reacted with 60 μM DPPH solution for 30 min in the dark, and absorbance was measured at 515 nm. Vitamin C was used as a positive control. Radical scavenging capacity was defined as the percentage (%) of the difference in absorbance between the sample and blank. We calculated the concentration of samples required to reduce the capacity for DPPH radical scavenging and DPPH radical absorbance by 50% (EC_50_). The maximum effective concentration was 50 nL/mL.

### 2.5. Analysis of Anti-Obesity Activities of CBVs

#### 2.5.1. Cell Culture

3T3-L1 cells purchased from the Korean Cell Line Bank (Seoul, Korea) were grown in DMEM containing 10% FBS and 1% penicillin/streptomycin until the cells reached confluence.

#### 2.5.2. Cell Viability Analysis

Cell viability was analyzed using the CCK-8 kit. 3T3-L1 cells were grown at a concentration of 1 × 10^4^ cells/well and treated with MV, CBV1, or CBV2 (1/2000, 1/1000, 1/500, and 1/100 dilution) for 24 h. After washing with DPBS, the cells were incubated with 20 µL of CCK-8 solution for 3 h. Absorbance was measured at 450 nm.

#### 2.5.3. Intracellular Triglyceride Analysis

We differentiated 3T3-L1 preadipocytes in MD1 media (DMEM containing 10% FBS, 0.5 mM IBMX, 1 μM dexamethasone, and 5 μg/mL insulin) for 2 days. Thereafter, 3T3-L1 adipocytes were incubated in MD2 media (10% FBS and 5 μg/mL of insulin) for 8 days. To measure intracellular triglycerides, 3T3-L1 adipocytes were incubated with 750 μL of solvent mixture (chloroform/methanol/H_2_O mixture, 8:4:3, *v*/*v*/*v*) at 37 °C for 60 min. After centrifugation at 4000× *g* at 4 °C for 10 min, the bottom organic layer was obtained and dried overnight. Extracted lipids were dissolved in 20 μL of ethanol and triglyceride levels were determined using an enzyme reaction kit (Asan Pharmaceutical, Seoul, Korea).

#### 2.5.4. Oil Red O (ORO) Staining and Quantification

To investigate the inhibition of lipid accumulation, ORO staining was performed. After fixation in 10% formalin for 30 min, 3T3-L1 adipocytes were washed with DPBS and stained with ORO solution for 15 min. Stained cells were captured and stained ORO in cells were dissolved with isopropanol to quantify. The absorbance was read at 510 nm.

#### 2.5.5. Anti-Obesity Related Biomarker Analysis (Real-Time PCR)

As mentioned above, 3T3-L1 preadipocytes were differentiated in MD1 media for 2 days with MV, CBV1, or CBV2 (1/100 dilution). Thereafter, 3T3-L1 adipocytes were incubated in MD2 media for 8 days. Total RNA was extracted using Trizol (Invitrogen, Carlsbad, CA, USA). cDNA was synthesized with 1 μg/mL total RNA using a TOPScript^TM^ cDNA synthesis kit. PCR was then performed with 10 μL of SYBR Green Premix, 0.5 μL of each primer (Supplementary Table S1), and 9 μL of cDNA (1/50 dilution). PCR amplification was conducted with enzyme activation at 94 °C for 10 min, followed by 45 cycles of denaturation at 94 °C for 15 s, and annealing and extension at 60 °C for 1 min each. The Ct value for each gene was normalized to that of GAPDH or β-actin.

### 2.6. Analysis of Anti-Aging Activities of CBVs

#### 2.6.1. Cell Culture

WI-38 cells, which are human normal embryonic lung-derived diploid fibroblasts (population doubling level, 23), were purchased from the Korean Cell Line Bank and grown in DMEM containing 10% FBS and 1% penicillin/streptomycin until reaching confluence.

#### 2.6.2. Cell Viability Analysis

WI-38 cells were grown at 1 × 10^4^ cells/well and treated with MV, CBV1, or CBV2 (1/2000, 1/1000, 1/500, and 1/100 dilution) for 24 h. To induce acute oxidative stress, WI-38 cells were pretreated with 50 μM H_2_O_2_ for 1 h. After washing with DPBS, the cells were incubated with 20 µL of CCK-8 solution for 3 h. Absorbance was measured at 450 nm.

#### 2.6.3. Cell Lifespan Analysis

Cell lifespan was evaluated by quantifying the population doubling level (PDL). WI-38 cells were grown at 1 × 10^5^ cells/well and treated with MV, CBV1, or CBV2 (1/100 dilution) for 24 h after pretreatment with 50 μM H_2_O_2_ for 1 h to induce acute oxidative stress. Each PDL was calculated as follows: current PDL = last PDL + log_2_ (collected cell number/seeded cell number).

#### 2.6.4. Anti-Aging-Related Biomarker Analysis (Real-Time PCR)

WI-38 cells at 2 × 10^5^ cells/well were treated with 50 μM H_2_O_2_ for 1 h, followed by treatment with MV, CBV1, or CBV2 (1/100 dilution) for 24 h. Total RNA was extracted from WI-38 cells using Trizol. cDNA was synthesized with 1 μg/mL total RNA using a TOPScript^TM^ cDNA synthesis kit. PCR was performed with 10 μL of SYBR Green Premix, 0.5 μL of each primer (Table 1) and 9 μL of cDNA (1/50 dilution). PCR amplification was conducted with enzyme activation at 94 °C for 10 min, followed by 45 cycles of denaturation at 94 °C for 15 s and annealing and extension at 60 °C for 1 min each. The Ct value for each gene was normalized with that of β-actin.

### 2.7. Statistical Analysis

Data are presented as the mean ± standard deviation (SD). Statistical significance was analyzed by one-way analysis of variance using GraphPad Prism 7 software (GraphPad, Inc., San Diego, CA, USA). *p* values < 0.05 were considered as statistically significant.

## 3. Results and Discussion

### 3.1. Physiochemical Characteristics of CBVs

**Figure 1:**
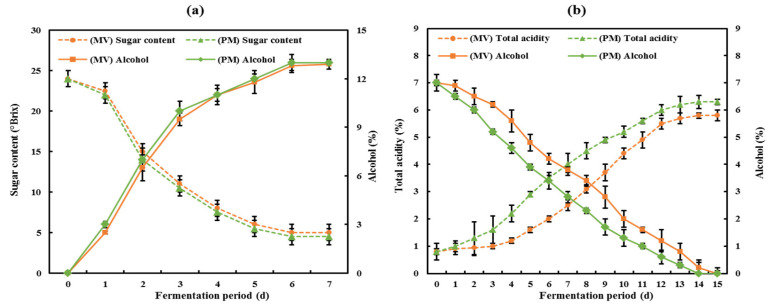
The two-stage fermentation for the production of MV and PMV vinegars. (**a**) Changes in sugar and alcohol content during alcohol fermentation. (**b**) Changes in alcohol content and total acidity during acetic acid fermentation. Data are expressed as mean ± SD.

Mandarin and premature mandarin wines showed similar fermentation rates, and the alcohol content was 12.8% and 13.0%, respectively (Figure 1a). During acetic acid fermentation, the fermentation rate of PMV was faster than that of MV, with 6% acidity reached on day 12. The final total acid content of PMV was 6.3%, which was higher than the 5.8% obtained for MV (Figure 1b). These results suggest that the acidity of the immature citrus juice samples was higher than that of the mature samples, which is similar to results reported by Yi et al. [18].

Evaluation of the physicochemical properties of CBVs revealed no significant difference in the pH of MV, CBV1, and CBV2, with an average value of 3.46 (Table 1). CBV2 showed the highest total acidity of 5.40% compared with MV (4.69%). The sugar content of MV was highest among the samples, at 68.40%. Song et al. reported that the sugar content increases as citrus fruits mature, which is closely related to the sweetness of fruit. In this study, the sugar content decreased when increasing amounts of PMV were added to CBV [19].

### 3.2. Antioxidant Activities of CBVs

**Figure 2:**
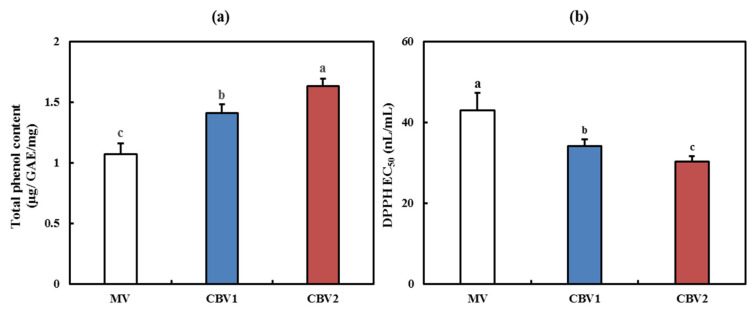
Antioxidant activities of citrus blended vinegars. (**a**) Total phenol content. (**b**) 2,2-diphenyl-1-picrylhydrazyl radical scavenging activity. Data are expressed as the mean ± SD. Lowercase letters indicate significant differences between groups (*p* < 0.05). MV—mandarin vinegar; CBV1—blended mandarin vinegar and premature mandarin vinegar in a ratio of 8:2; CBV2—blended mandarin vinegar and premature mandarin vinegar in a ratio of 7:3.

Polyphenolic compounds, such as flavonoids, anthocyanin, tannin, and catechin, are widely distributed in plants such as fruits and leafy vegetables. Phenolic compounds include phenols, phenolic acids, flavonoids, and phenylpropanoids, which show anti-allergic, anti-bacterial, antioxidant, and hyperlipidemic properties [20]. The TPC of CBVs was shown to be significantly higher than that of MV (Figure 2a, *p* < 0.05). Particularly, the TPC of CBV2 showed the highest value at 1.63 μg GAE/mL, which was 1.5-fold greater than that of MV. According to previous studies, the chemical content of the raw material used to prepare vinegar influences the TPC of the vinegar [21,22]. TPC was higher than our results; however, similar to our results, TPC of vinegar with immature Citrus unshiu showed higher value than that of vinegar with mature Citrus ushiu [21]. In this study, the high TPC value of CBVs increased with the addition of PMV.

As shown in Figure 2b, the EC_50_ values were lower than that of vitamin C in all samples. Particularly, CBV2 showed the lowest EC_50_ value at 30.22 nL/mL. Accordingly, the EC_50_ values of CBV1 and CBV2 were significantly higher than those of MV (*p* < 0.05), indicating higher antioxidant activity. According to Ousaaid et al., the DPPH value of apple vinegar was higher than that of our result at 0.74 ± 0.154 μL/mL [23]. As previously reported, immature citrus fruits contain more organic acids, polyphenols, and flavonoids than mature fruits [18]. Notably, the pericarp contains larger quantities of physiologically active ingredients, such as essential oils, carotenoids, and flavonoids [12]. Additionally, the high TPC in fruits has been reported to be closely related to their DPPH radical scavenging activity [24,25]. Consistent with previous reports [25], CBVs with high TPC exhibited high DPPH radical scavenging activity. Based on these results, CBVs with a high PMV content may have high antioxidant activity.

### 3.3. Anti-Obesity Activities of CBVs

**Figure 3:**
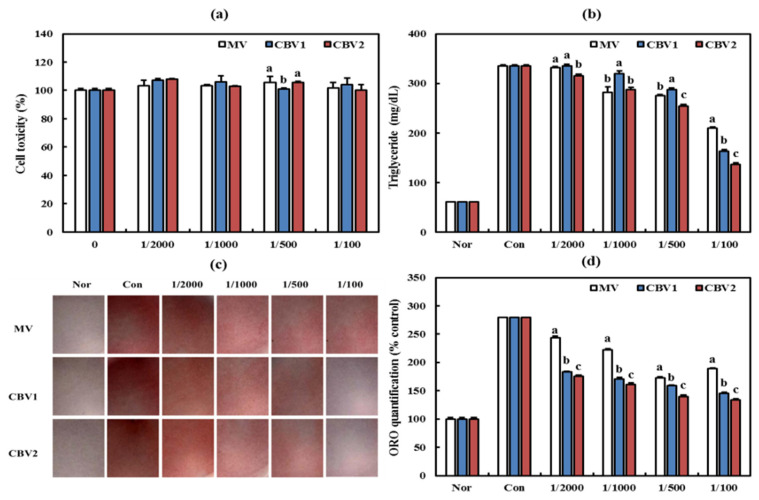
Anti-obesity activities of CBVs in 3T3-L1 cells. (**a**) Cytotoxicity. (**b**) Concentration of intracellular triglycerides. (**c**) Oil red O staining. (**d**) Quantification of Oil red O staining. Data are expressed as the mean ± SD. Lowercase letters indicate significant differences between groups (*p* < 0.05). MV—mandarin vinegar; CBV1—blended mandarin vinegar and premature mandarin vinegar in a ratio of 8:2; CBV2—blended mandarin vinegar and premature mandarin vinegar in a ratio of 7:3.

As shown in Figure 3a, cell viability was higher than 96%, indicating low cytotoxicity in all experimental groups. Similarly, Park et al. found that lyophilized dropwort vinegar powder had low cytotoxicity in 3T3-L1 cells [26].

The intracellular triglyceride concentration in all CBVs decreased in a dose-dependent-manner (Figure 3b). Specifically, the triglyceride concentration of CBV2 at 1/100 dilution was the lowest at 136.12 mg/dL, which was 65% that of MV (209.82 mg/dL). The triglyceride-lowering effect of various vinegars in 3T3-L1 cells has been reported previously [27,28]. According to Lee et al., tomato vinegar significantly reduces the triglyceride content by 45.71% compared with controls (*p* < 0.01) [27]. These results indicate that the proper blending of citrus vinegar can lower triglyceride levels, promoting anti-obesity effects.

In ORO results, CBVs significantly inhibited lipid accumulation in 3T3-L1 adipocytes (Figure 3c). Similarly, Figure 3d shows the dose dependency of CBVs and significantly decreased number of ORO-stained cells compared with that in controls (*p* < 0.05). Consistent with our triglyceride results, CBV2 at 1/100 dilution showed strong inhibition of lipid accumulation compared with MV. Similarly, Son et al. reported that spirit vinegar and natural fermented vinegar products strongly inhibit lipid accumulation in a dose-dependent manner [29]. These results demonstrate the anti-obesity activities of CBVs in 3T3-L1 cells.

**Figure 4:**
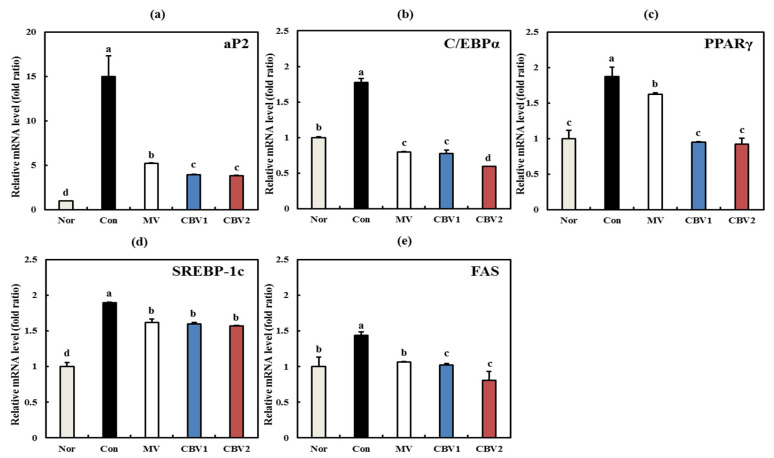
Anti-obesity related gene expression of CBVs in 3T3-L1 cells. (**a**) aP2 mRNA level; (**b**) C/EBP mRNA level; (**c**) PPARγ mRNA level; (**d**) SREBP-1c mRNA level; (**e**) FAS mRNA level. Data are expressed as the mean ± SD. Lowercase letters indicate significant differences among groups (*p* < 0.05). Nor—not differentiated; Con—differentiated; MV—differentiated + mandarin vinegar; CBV1—differentiated + blended mandarin vinegar and premature mandarin vinegar in a ratio of 8:2; CBV2—H_2_O_2_ treated + blended mandarin vinegar and premature mandarin vinegar in a ratio of 7:3.

To confirm the anti-obesity effect of CBVs, adipogenic and lipogenic genes associated with obesity were measured using quantitative real-time PCR. According to the previous studies, adipocyte fatty acid binding protein (*aP2*), CCAAT/enhancer binding protein *α* (*C*/*EBPα)*, and peroxisome proliferator-activated receptor γ (*PPARγ*) are known to induce adipogenic differentiation gene expression as transcription factors [30], while sterol regulatory element binding protein (*SREBP-1c*) and fatty acid synthase (*FAS*) are involved in lipogenic differentiation [31]. As shown in Figure 4a–c, the mRNA expression of adipogenic genes, such as *aP2*, *C*/*EBPα*, and *PPARγ* was significantly downregulated by CBV treatment (*p* < 0.05). Similarly, Hosoda et al. reported that ginkgo vinegar significantly decreased *C*/*EBPα* and *PPARγ* expression [28]. Additionally, Figure 4d–e shows that the mRNA expression levels of the lipogenic genes, sterol regulatory element binding protein (*SREBP-1c*), and fatty acid synthase (*FAS*) were also significantly decreased following CBV treatment. Similar to previous studies, [27,28,29] our results show that CBVs reduced the expression of adipogenic genes and lipogenic genes. These results confirm the anti-obesity activities of CBVs via controlling adipogenic and lipogenic gene levels in 3T3-L1 cells.

### 3.4. Anti-Aging Activities of CBVs

**Figure 5:**
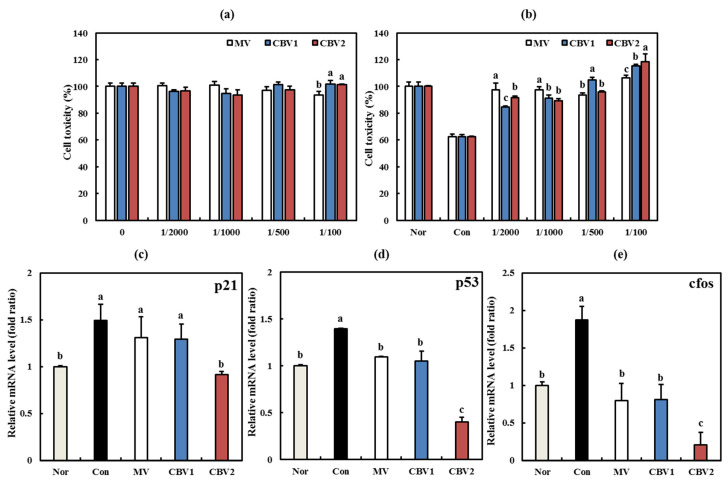
Anti-aging activities of citrus blended vinegars in WI-38 cells. (**a**) Cytotoxicity. (**b**) Cytotoxicity in H_2_O_2_-induced cells. (**c**) p21 mRNA level. (**d**) p53 mRNA level; and (**e**) c-Fos mRNA level. Data are expressed as the mean ± SD. Lowercase letters indicate significant differences among groups (*p* < 0.05). Nor—H_2_O_2_ not treated; Con—H_2_O_2_ treated; MV—H_2_O_2_ treated + mandarin vinegar; CBV1—H_2_O_2_ treated + blended mandarin vinegar and premature mandarin vinegar in a ratio of 8:2; CBV2—H_2_O_2_ treated + blended mandarin vinegar and premature mandarin vinegar in a ratio of 7:3.

CBVs did not exhibit cytotoxicity in any of the groups (Figure 5a), which is consistent with the cell cytotoxicity results in 3T3-L1 cells. Rather, CBVs exhibited cell-protective effects in H_2_O_2_-induced WI-38 cells by increasing the reduced cell viability following treatment with H_2_O_2_ (Figure 5b, *p* < 0.05). CBV2 at a 1/100 dilution increased cell viability to 118%. Similarly, a previous study reported that treatment with H_2_O_2_ decreased cell survival by 43%, while caffeic acid elicited a cell-protective effect by increasing the cell survival rate [32]. In another study, treatment with H_2_O_2_ was shown to decrease cell viability by up to 70%, which was reversed following administration of porphyrin, which promoted cell viability [33]. These results indicate that CBV2 protects cells against acute oxidative stress conditions without causing cell toxicity. 

CBVs were also observed to increase the cell lifespan in H_2_O_2_-induced WI-38 cells (Table 2). Cell lifespan with H_2_O_2_ was decreased from PDL 24 to PDL 19. However, CBV2 recovered cell liver span to PDL 27. Similarly, malvidin recovered the reduced cell lifespan upon H_2_O_2_ treatment in all age groups [34]. These results indicate that CBVs shows anti-aging effects by recovering cell lifespan in acute oxidative stress conditions.

To confirm the anti-aging activity of CBVs (1/100 dilution), we measured the mRNA levels of the aging-related genes p21, p53, and c-Fos in H_2_O_2_-induced WI-38 cells. H_2_O_2_ led to increased levels of aging-related markers, whereas CBVs significantly reduced these levels (Figure 5c–e, *p* < 0.05). Particularly, CBV2 strongly inhibited the mRNA expression of p21, p53, and c-Fos, all of which are known markers of cellular senescence. The p21 gene plays a pivotal role in the senescent phenotype of human fibroblasts [35]. Similarly, activation of p53 has also been reported to induce cellular senescence [36]. Meanwhile, the expression of c-Fos, an early response gene, has been shown to be decreased in fibroblast senescence [37]. According to Seo and colleagues, malvidin exerts anti-aging effects by reducing the protein expression of p21 and p53 [35]. In another report, the acute stress inducer UVB increased the mRNA levels of p21, p53, and c-Fos, similar to treatment with H_2_O_2_ [38]. Based on these results, we confirmed that the anti-aging properties of CBV2 occur through its cell-protective effect.

## 4. Conclusions

In this study, we prepared CBVs by blending two citrus vinegars (MV, PMV). While the pH values of the CBVs were similar, the amount of PMV added correlated with higher acidity and lower sugar content. Moreover, the antioxidant activities of CBVs, along with TPC, were higher than those of MV. Additionally, CBV was observed to elicit anti-obesity activities via reducing the intracellular triglyceride content, lipid accumulation, and mRNA levels of adipogenic and lipogenic-related genes in 3T3-L1 cells. Still further, the anti-aging activities of CBVs were confirmed in WI-38 cells by increasing cell viability and decreasing the mRNA levels of *p21*, *p53*, and *c-Fos* under conditions of H_2_O_2_-induced oxidative stress. Taken together, CBVs showed strong antioxidant, anti-obesity, and anti-aging effects, indicating their nutritional value.

## Figures and Tables

**Table 1:** Physicochemical characteristics of citrus blend vinegar.

Characteristic	MV	CBV1	CBV2
pH	3.45 ± 0.11 ^a^	3.37 ± 0.40 ^a^	3.46 ± 0.19 ^a^
Total acidity (%)	4.69 ± 0.11 ^b^	5.04 ± 0.20 ^ab^	5.40 ± 0.22 ^a^
Sugar content (%)	68.40 ± 0.00 ^a^	54.93 ± 0.23 ^b^	53.60 ± 0.00 ^c^

Data are expressed as the mean ± SD. Lowercase letters indicate significant differences among groups (*p* < 0.05).

**Table 2:** Population doubling level (PDL) of citrus blended vinegars.

H_2_O_2_	H_2_O_2_ Plus MV	H_2_O_2_ Plus CBV1	H_2_O_2_ Plus CBV2
24 19	24 22	24 26	24 27

PDL—last PDL + log_2_ (collected cell number/seeded cell number).

## Data Availability

The data presented in this study are available in the article.

## Supplementary Materials

The following are available online at https://www.mdpi.com/article/10.3390/foods10071441/s1, Table S1: Primer sequences used in quantitative real-time PCR.
