# Supplementary material for: Antioxidant, Anti-Obesity, and Anti-Aging Activities of Jeju Citrus Blended Vinegar
Source: Foods. 2021 Jun 22;10(7):1441. doi: 10.3390/foods10071441 (PMC8306124; doi:10.3390/foods10071441)
Supplement: Supplementary file 1 [file foods-10-01441-s001.zip › foods-1247658-supplementary.pdf]

**Table S1.** Primer sequences used in quantitative real-time PCR

| Gene | Forward primer (5′-3′) | Reverse primer (5′-3′) |
|---|---|---|
| **Mouse** | | |
| GAPDH | GTATGACTCCACTCACGGCAAA | GGTGTGGCTCCTGGAAGATG |
| aP2 | CATGGCCAAGCCCAACAT | CGCCCAGTTTGAAGGAAATC |
| C/EBPα | AGGTGCTGGAGTTGACCAGT | CAGCCTAGAGATCCAGCGAC |
| PPARγ | CGCTGATGCACTGCCTATGA | AGAGGTCCACAGAGCTGATTCC |
| SREBP-1c | AGAGGGTGAGCCTGACAA | CCTCTGCAATTTCCAGAT |
| FAS | CTGAGATCCCAGCACTTCTTGA | GCCTCCGAAGCCAAATGAG |
| **Human** | | |
| β-actin | CCACCATGTACCCTGGCATT | CGGACTCGTCATACTCCTGC |
| p21 | TTCTCCACCTAGACTGTAA | GCACCTGCTGTATATTCA |
| p53 | GTGTGGAGTATTTGGATGAC | ATGTAGTTGTAGTGGATGGT |
| c-Fos | TCACCCGCAGACTCCTTCTC | GTGGGAATGAAGTTGGCACTG |
